# The Effects of Fe_2_O_3_ Nanoparticles on Physiology and Insecticide Activity in Non-Transgenic and Bt-Transgenic Cotton

**DOI:** 10.3389/fpls.2015.01263

**Published:** 2016-01-22

**Authors:** Le Van Nhan, Chuanxin Ma, Yukui Rui, Weidong Cao, Yingqing Deng, Liming Liu, Baoshan Xing

**Affiliations:** ^1^College of Resources and Environmental Sciences, China Agricultural UniversityBeijing, China; ^2^Center for Training, Consultancy, and Technology Transfer, Vietnam Academy of Science and TechnologyHanoi, Vietnam; ^3^Stockbridge School of Agriculture, University of Massachusetts Amherst, AmherstMA, USA; ^4^Institute of Resource and Regional Planning, Chinese Academy of Agricultural SciencesBeijing, China

**Keywords:** fate, phytotoxicity, Fe_2_O_3_ nanoparticles, insecticide activity, Bt-transgenic cotton

## Abstract

As the demands for nanotechnology and nanoparticle (NP) applications in agriculture increase, the ecological risk has drawn more attention because of the unpredictable results of interactions between NPs and transgenic crops. In this study, we investigated the effects of various concentrations of Fe_2_O_3_ NPs on Bt-transgenic cotton in comparison with conventional cotton for 10 days. Each treatment was conducted in triplicate, and each experiment was repeated three times. Results demonstrated that Fe_2_O_3_ NPs inhibited the plant height and root length of Bt-transgenic cotton and promoted root hairs and biomass of non-transgenic cotton. Nutrients such as Na and K in Bt-transgenic cotton roots increased, while Zn contents decreased with Fe_2_O_3_ NPs. Most hormones in the roots of Bt-transgenic cotton increased at low Fe_2_O_3_ NP exposure (100 mg⋅L^-1^) but decreased at high concentrations of Fe_2_O_3_ NPs (1000 mg⋅L^-1^). Fe_2_O_3_ NPs increased the Bt-toxin in leaves and roots of Bt-transgenic cotton. Fe_2_O_3_ NPs were absorbed into roots, then transported to the shoots of both Bt-transgenic and non-transgenic cottons. The bioaccumulation of Fe_2_O_3_ NPs in plants might be a potential risk for agricultural crops and affect the environment and human health.

## Introduction

Iron oxide (Fe_2_O_3_), the most common oxide of iron, has important magnetic properties. Iron (III) oxide is a convenient compound for the general study of polymorphism and the magnetic and structural phase transitions of NPs. Fe_2_O_3_ NPs can be applied in the fields of photoelectrochemistry (such as solar energy conversion and water splitting) and photocatalysts for the removal of organic and inorganic species from aqueous or gas phases (Chirita and Grozescu, 2009). Ali et al. (2012) reported that the addition of Fe_2_O_3_ NPs in cement could improve the strength and water permeability of the specimens. Introducing Fe_2_O_3_ NPs into soil could significantly increase root elongation and photosynthesis rate in soybean as compared with bulk Fe_2_O_3_. Similar results have also been reported for rice seedlings (*Oryza sativa L.* var. *Koshihikari*) treated with 500, 1000, and 2000 mg⋅L^-1^γ-Fe_2_O_3_ NPs when compared with the control and bulk treatments (Alidoust and Isoda, 2014). Thus, nano-size effects might enhance plant growth relative to bulk metal. With the development of nanotechnology, a rapidly growing body of concerns has been raised regarding the potential risks and negative effects of NPs on the environment and human health.

The toxicity of metal NPs is not clearly known to living organisms, but previous studies have shown that nanotoxicity is generally affected by unique properties (particle size, shape, and surface properties) (Crane et al., 2008; Navarro et al., 2008). Nanotoxicity has been tested using various organisms, including bacteria, algae, protozoa, plants, and fish (U.S. EPA, 1993). The metabolic processes in living organisms have been investigated to assess its toxicity to the environment (Bitton, 1999). However, in the plant kingdom, investigation of phenotypic differences, seed germination, and plant biomass in response to NP exposure could be one of the most effective ways to assess NP toxicity (Wang and Liu, 2001; Boutin et al., 2004; Di Salvatore M, 2008; Parsons et al., 2010). For example, alumina (Al_2_O_3_) NPs can cause phytotoxicity by inhibiting root elongation in corn, cucumber, soybean, cabbage, and carrot (Yang and Watts, 2005). This phytotoxicity is also evident for other plant species, including radish, rape canola, ryegrass, lettuce, corn, and cucumber, when treated with multiwall carbon nanotubes, aluminum (Al), alumina (Al_2_O_3_), zinc (Zn), and zinc oxide (ZnO) NPs (Lin and Xing, 2007). In addition to NP-induced toxicity in plants, different plant species could respond differently in the same NP exposure. In a study by Li et al. (2014), CeO_2_ NPs were revealed to have toxic effects on the root biomass of Bt-transgenic cotton under 100 and 500 mg⋅L^-1^ exposures, but to have no effects on conventional cotton. In addition, Le et al. (2014) reported that SiO_2_ NPs negatively affected the activities of CAT in the roots of both Bt-transgenic and non-transgenic cottons.

Insect pests can significantly reduce crop yields and subsequently cause economic losses in agriculture all over the world. At present, chemical pesticides are still the major method to control pest damage; however, the disadvantages of chemical pesticide usage in agriculture include the fact that pests becomes resistant to the chemicals and overuse of pesticides can cause serious risks to the environment and human health (Liu et al., 2015). As such, transgenic plants have been widely applied in agriculture for the purpose of controlling pest damage. For example, over expression of *Bacillus thuringiensis* (Bt) insecticidal protein in cotton can significantly enhance the resistance of cotton to insects and control insect damage (Wang, 2007; Huang et al., 2010; Yu et al., 2014). Currently, Bt-transgenic cotton is widely used in agriculture, and China is the largest cotton producing country in the world (Du, 2001). However, as demand for nanotechnology and NP applications in agriculture increases, the ecological risks are drawing attention because of the unpredictable results in interactions between NPs and transgenic crops.

To our knowledge, this is the first study on the effects of Fe_2_O_3_ NPs on Bt-transgenic cotton. In this study, the toxic effects of Fe_2_O_3_ NPs on both conventional and Bt-transgenic cottons were investigated from the aspects of plant growth, nutrient levels, hormone levels, and the changes to Bt-toxic protein in Bt-transgenic cotton in the presence of Fe_2_O_3_ NPs. Fe_2_O_3_ NP accumulation and distribution were also assayed to reveal how NPs are distributed inside cotton. TEM images showed that Fe_2_O_3_ NPs were found in the roots of both conventional and transgenic cotton.

## Materials and Methods

### Characterization of Fe_2_O_3_ Nanoparticles

Fe_2_O_3_ NPs were purchased from Shanghai Hufeng Bioscience Technology Company (Shanghai City, China). Scanning electron microscopy (JEOL JSM 5600, Japan) was used to determine the morphology of Fe_2_O_3_ NPs. TEM images were obtained (JEM 200CX, Japan) at 200 kV. Following Amrut et al. (2010), the samples were prepared by dispersing drops of the colloid on a copper grid, which was then covered with a carbon film, and the solvent was evaporated. Fe_2_O_3_ NP suspensions were prepared at a concentration of 2 mg⋅L^-1^ for measurement of hydrodynamic size and zeta potential (Nicomp 380 DLS Zeta potential/Particle system, Santa Barbara, CA, USA).

### Experimental Exposure

Bt-transgenic cotton (Bt-29317) and conventional cotton (Jihe 321) were purchased from the Chinese Academy of Agricultural Sciences, China Agricultural University. Four cotton plants were allowed to acclimatize in a pot containing 2.0 L of nutrient solution for 4 days and then exposed to 0, 100, or 1000 mg⋅L^-1^ Fe_2_O_3_ NPs for 10 days. The nutrient solution was made following previous reports by Li et al. (2014). Each treatment was performed in triplicate, and each experiment was repeated three times. Fe_2_O_3_ NPs were dispersed in DI H_2_O for 30 min using an ultra-sonicator (KQ3200DE) (Le et al., 2014; Li et al., 2014). The experiments were conducted in a greenhouse with natural light, humidity, and temperature at China Agricultural University.

### Measurement of Biomass, Plant Height, Root Length, and Root Hairs

The plant height (mm) and root length (mm) were measured from the growth point in shoot to the cotyledon node and from the growth point to the root point, respectively, after 10 days of exposure to Fe_2_O_3_ NPs (Le et al., 2014). The root hairs (root) were counted one by one in each cotton plant.

The samples were collected at day 10. The shoots and roots were rinsed with both tap water and DI H_2_O for 5 min three times and then dried separately at 80°C for 24–36 h until a constant dry weight was obtained. The dry weight was used to determine the effects of the Fe_2_O_3_ NPs on plant growth.

### Measurement of Nutrient Contents

Nutrient contents were determined using inductivelycoupled plasma (ICP) mass spectrometry (DRC-II) and ICP atomic emission spectroscopy (iCap 6000) (Li et al., 2014). Fine powders (20–30 mg) of oven-dried shoots and roots were digested in 5 mL of HNO_3_ at room temperature for 24 h; then, 3 mL of H_2_O_2_ was added to each sample to further digest samples at 180°C for 4–5 h.

### Determination of Hormone Concentration

Extraction and purification of ABA, IAA, ZR, and GA were performed with enzyme-linked immune absorbent assay (ELISA) kits using monoclonal antibodies (Phytodetek, Agdia, Elkhart, IN, USA) as described in He et al. (2005) and Dong et al. (2008). All samples were measured at 405 nm after the purification step (Wang et al., 2012).

### Determination of Bt Toxin

Approximately 0.5-g samples of roots and leaves of Bt-transgenic cotton were harvested and stored at -40°C until analysis. The samples were placed in 9.5 mL of extraction solution (1.33 g of Na_2_CO_3_, 1.46 g of NaCl, 0.5 g of vitamin C, and 0.25 g of dithiothreitol in 250 mL of DI H_2_O). The mixture was gently shaken for 30 min before being centrifuged at 4,000 rpm for 15 min. The supernatants were used to analyze the levels of the Bt toxin protein using an ELISA procedure (Rui et al., 2005).

### Transmission Electron Microscopy Observation

Fe_2_O_3_ NP-treated shoot and root samples of both conventional and Bt-transgenic cotton were harvested and washed with DI H_2_O at day 10. Each sample was prefixed in 2.5% glutaraldehyde, then washed in 0.1 mol⋅L^-1^ pH 7.0 phosphate buffer mixed with 1% osmium tetroxide for 2 h before being dehydrated in a graded ethanol series and finally embedded in epoxy resin. The samples for TEM observation were sectioned using the procedure described in Zhang et al. (2012).

### Data Analysis

The results are presented as the mean ± SD. One-way analysis of variance was used to calculate statistical analysis (SPSS 22.0 software). A confidence interval of 95% (*p* < 0.05) was considered significant in all cases.

## Results And Discussion

### Characterizations of Fe_2_O_3_ NPs

**FIGURE 1:**
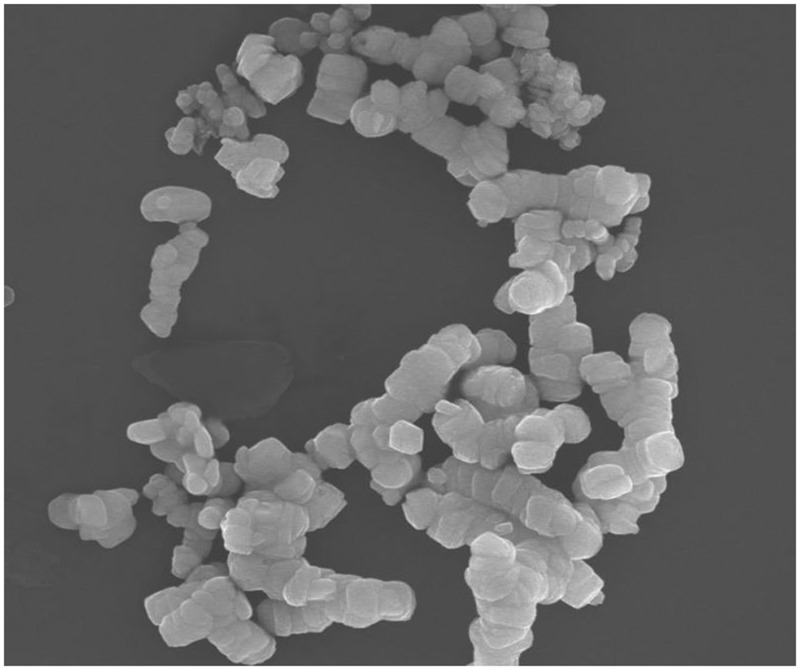
**SEM images of Fe_2_O_3_ NPs**.

Fe_2_O_3_ NPs were purchased from Sigma Inc. The advertised diameter was smaller than 50 nm, the specific surface area was from 50 from 245 m^2^g^-1^, and the density was 5.25 g⋅cm^3^; the crystal phase was cubic. Fe_2_O_3_ NPs were characterized by scanning electron microscopy (JEOL JSM 5600, Japan) and dynamic light scattering (DLS). In addition, Fe_2_O_3_ NPs were prepared at concentration of 2 mg⋅L^-1^ for measurement of hydrodynamic size (154.3 nm) and zeta potential (-9.27 mV; Nicomp 380 DLS Zeta potential/Particle system, Santa Barbara, CA, USA). **Figure 1** gives the SEM image; the diameter was larger than the advertised data.

### Effects of Fe_2_O_3_ NPs on the Growth of Cotton Plants

**FIGURE 2:**
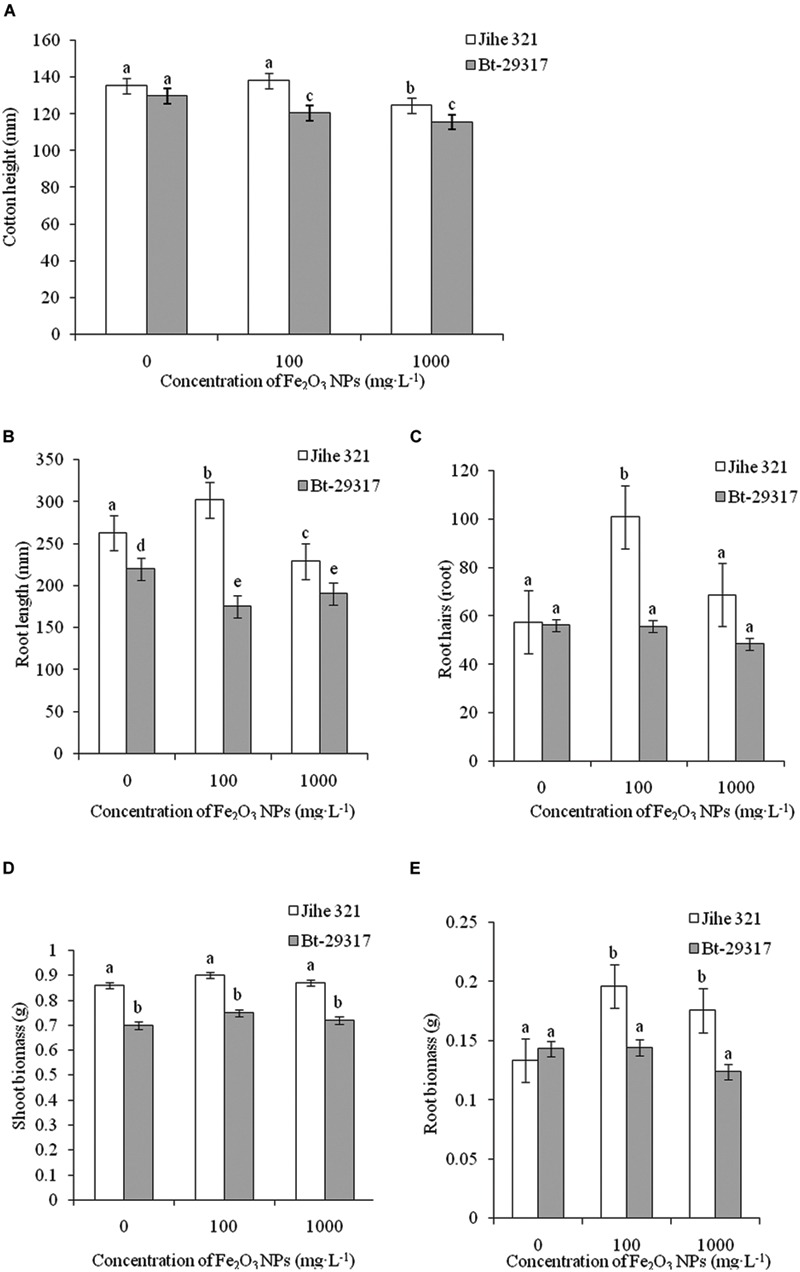
**Effects of Fe_2_O_3_ NPs on the growth of cotton plants. (A–E)** Represents plant height, root length, root hairs, shoot biomass, and root biomass, respectively. The values are presented as means ± SD. Different small letters for the same cultivar indicate a significant difference at *p* < 0.05 level between control and Fe_2_O_3_ NP exposures, and different small letters for the same Fe_2_O_3_ NP concentration indicate a significant difference at *p* < 0.05 level between Bt-transgenic and non-transgenic cotton.

In control groups, no significant difference in plant height was observed between conventional and Bt-transgenic cotton. However, a decrease in plant height for Bt-transgenic cotton was shown with both 100 and 1000 mg⋅L exposure doses of Fe_2_O_3_ NPs, while similar phenotypic difference was only observed in 1000 mg⋅L Fe_2_O_3_ NP-treated conventional cotton (**Figure 2A**). Although root length in conventional cotton is approximately 12.5% longer than in Bt-transgenic cotton without the Fe_2_O_3_ NP exposure, a significant decrease in the root length in 100 mg⋅L Fe_2_O_3_ NP-treated transgenic cotton was evident. The opposite phenomenon in Fe_2_O_3_ NP-treated conventional cotton was attained (**Figure 2B**). Similarly, a low exposure dose of Fe_2_O_3_ NPs could stimulate development of root hairs in conventional cotton as compared with its respective control group, while no difference was found in transgenic cotton among the treatments in **Figure 2C**. Regarding fresh biomass of separated shoots and roots in both types of cottons, shoot biomass in conventional cotton was similar among treatments; however, root biomass in Fe_2_O_3_ NP-treated conventional cotton was 30.8–41.2% higher relative to its control group (**Figures 2D,E**). Analysis of shoots and roots biomass in Bt-transgenic cotton showed that Fe_2_O_3_ NPs seemed to have no impact on biomass. These results suggest that Bt-transgenic cotton is more sensitive to NP exposure than conventional cotton. A similar result was reported by Li et al. (2014), in which 100 and 500 mg⋅L^-1^ CeO_2_ NPs could cause more toxicity to Bt-transgenic cotton by reducing root biomass, while no impact was found in non-transgenic cotton. However, phytotoxicity might be dependent on NP species. For example, as SiO_2_ NP concentration increased up to 2000 mg⋅L^-1^, the plant height and biomasses of both conventional and Bt-transgenic cottons significantly decreased (Le et al., 2014). Other NPs could also impact plant biomass, such as ZnO and CuO NPs. Ryegrass (*Loliumperenne*) biomass was significantly reduced by different concentrations (0–1000 mg⋅L^-1^) of ZnO NPs. Wang et al. (2015) demonstrated that the root length and biomass of the transgenic rice *O. sativa* (OsCDC2 and OsCYCD genes, which play important roles in controlling the cell cycle during plant development) were inhibited by 5 mg⋅L^-1^ CuO NPs and 5 mg⋅L^-1^CuO bulk particles after 72-h exposure.

### Effects of Fe_2_O_3_ NPs on Nutrient Contents in Cotton Plants

**FIGURE 3:**
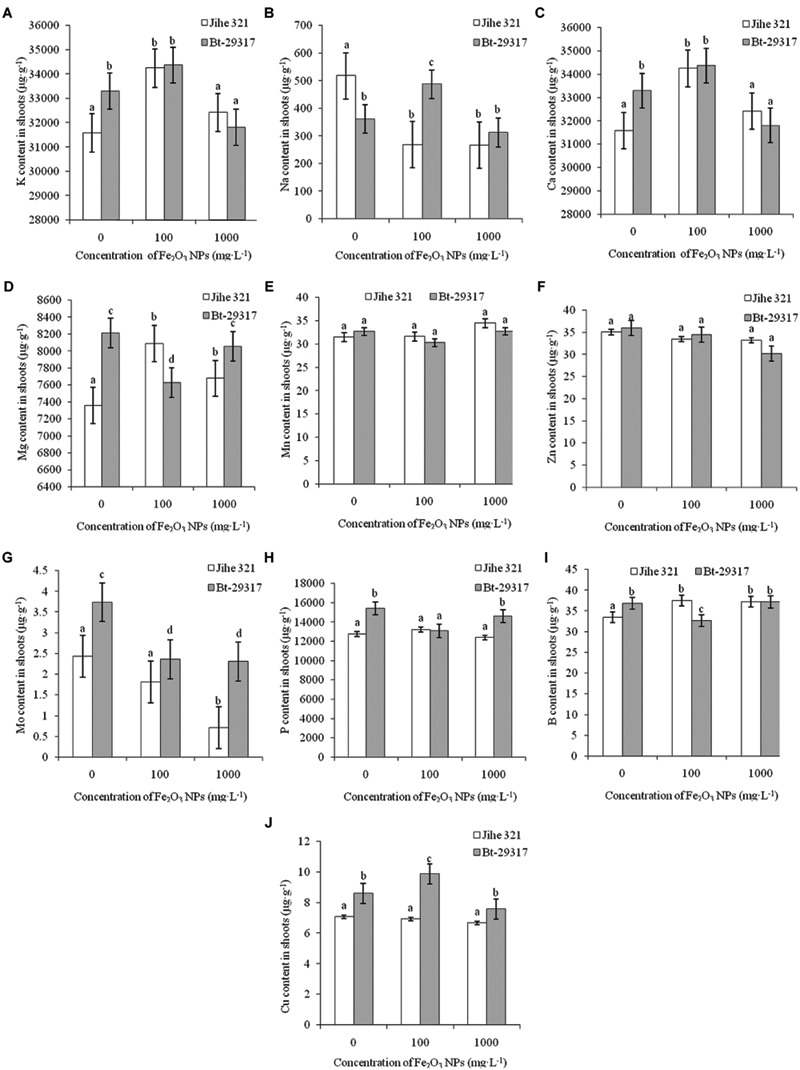
**Effects of Fe_2_O_3_ NPs on nutrient contents in the shoots of cotton plants. (A–J)** Shows the levels of K, Na, Ca, Mg, Mn, Zn, Mo, P, B, and Cu in Fe_2_O_3_ NPs treated shoots of the conventional and the Bt-transgenic cottons, respectively. The values are presented as means ± SD. Different small letters for the same cultivar indicate a significant difference at *p* < 0.05 level between control and Fe_2_O_3_ NP exposures, and different small letters for the same Fe_2_O_3_ NP concentration indicate a significant difference at *p* < 0.05 level between Bt-transgenic and non-transgenic cotton.

Nutrient contents (macronutrients and micronutrients) in both types of cotton treated by Fe_2_O_3_ NPs were investigated. K is one of the most important micro nutrients in plants. K contents in both types of cottons were increased in the 100 mg⋅L^-1^ Fe_2_O_3_ NP treatment, although this increase was not statistically significant in Bt-transgenic cotton compared with its corresponding control (**Figure 3A**). Similar trends were also observed for Ca contents in shoots (**Figure 3C**). Fe_2_O_3_ NPs inhibited Na uptake in shoots of conventional cotton at both 100 mg⋅L^-1^ and 1000 mg⋅L^-1^, whereas an increase in Na in the shoots was found for 100 mg⋅L^-1^ Fe_2_O_3_ NP-treated Bt-transgenic cotton (**Figure 3B**). Cotton type could also determine the nutrient content. For example, in the absence of Fe_2_O_3_ NPs, Mg content in conventional cotton was approximately half that of transgenic cotton. When treated with various concentrations of Fe_2_O_3_ NPs, increases in Mg were observed in conventional cotton shoots treated with Fe_2_O_3_ NPs, whereas a decrease in Mg in 100 mg⋅L Fe_2_O_3_ NP-treated Bt-transgenic shoots was found (**Figure 3D**). Similar results were also found in B content in both conventional and transgenic cottons (**Figure 3I**). Fe_2_O_3_ NPs significantly decreased Mo contents in shoots of both conventional and Bt-transgenic cottons (**Figure 3G**). Other elemental contents, including Mn, Zn, P, and Cu (**Figures 3E,F,H,J**), showed no change in either type of plant in the presence of Fe_2_O_3_ NPs. These results suggest that Fe_2_O_3_ NPs could more seriously interrupt nutrient uptake in Bt-transgenic cotton as compared with conventional cotton.

**FIGURE 4:**
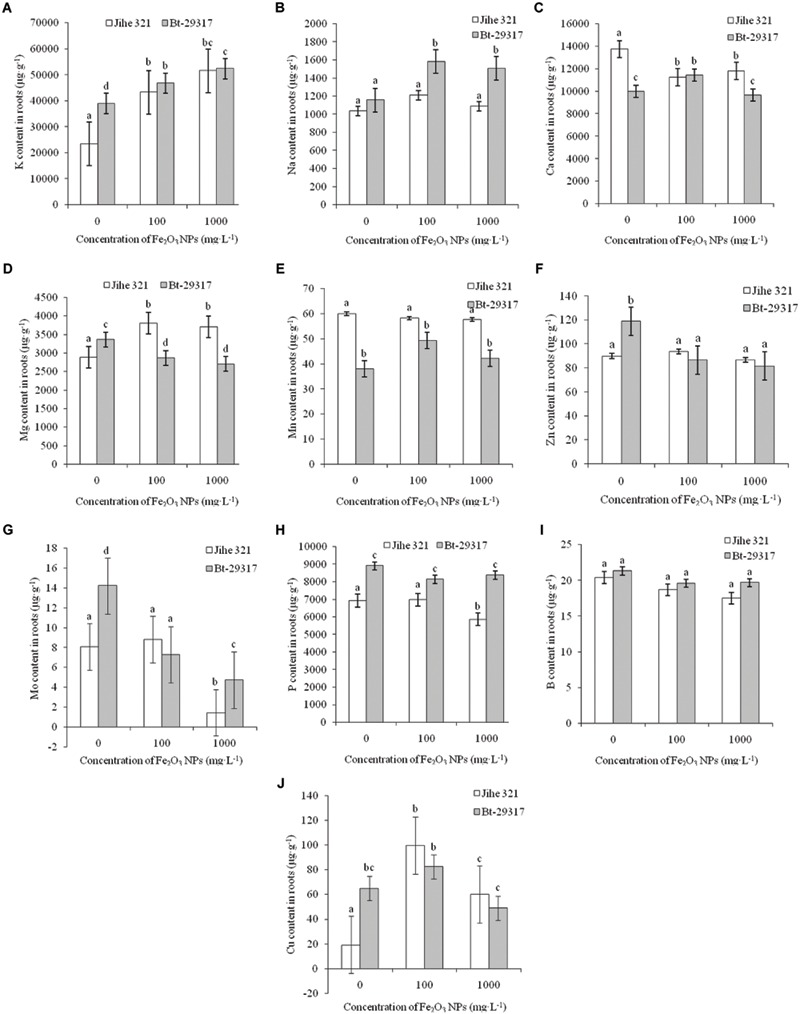
**Effects of Fe_2_O_3_ NPs on nutrient contents in the roots of cotton plants. (A–J)** Shows the levels of K, Na, Ca, Mg, Mn, Zn, Mo, P, B, and Cu in Fe_2_O_3_ NPs treated roots of the conventional and the Bt-transgenic cottons, respectively. The values are presented as means ± SD. Different small letters for the same cultivar indicate a significant difference at *p* < 0.05 level between control and Fe_2_O_3_ NP exposures, and different small letters for the same Fe_2_O_3_ NP concentration indicate a significant difference at *p* < 0.05 level between Bt-transgenic and non-transgenic cotton.

**Figure 4** shows the trend of nutrient uptake in roots of both types of cotton. Fe_2_O_3_ NPs significantly enhanced K uptake in the roots of both Bt-transgenic and non-transgenic cotton (**Figure 4A**). A significant increase in Na content was only observed in the roots of Bt-transgenic cotton in **Figure 4B**. Fe_2_O_3_ NPs only caused a decrease in Ca uptake in conventional cotton, whereas 100 mg⋅L^-1^ Fe_2_O_3_ NPs enhanced Ca content in Bt-transgenic root (**Figure 4D**). The opposite results were found for Mg content in both types of cotton in **Figure 4D**. In conventional cotton, Fe_2_O_3_ NPs significantly stimulated Mg uptake at both concentrations, while a decrease in Mg content was evident for Bt-transgenic cotton relative to its corresponding control. No significant effects of Fe_2_O_3_ NPs were seen for Mn content in roots, regardless of type of cotton, although conventional cotton could take up more Mn than Bt-transgenic cotton in the absence of Fe_2_O_3_ NPs (**Figure 4E**). Fe_2_O_3_ NPs decreased the Zn content in the roots of Bt-transgenic cotton, while no significant difference was reported in Zn content for conventional cotton roots (**Figure 4F**). The presence of 100 mg⋅L^-1^ of Fe_2_O_3_ NPs could enhance Cu uptake in the roots of both types of cottons, and Cu contents in both plants slightly decreased at 1000 mg⋅L^-1^ (**Figure 4J**). Fe_2_O_3_ NPs decreased Mo contents in the roots of both conventional and Bt-transgenic cottons at 1000 mg⋅L^-1^ (**Figure 4G**). P contents were not affected by Fe_2_O_3_ NPs (**Figure 4H**), except for 1000 mg⋅L^-1^ Fe_2_O_3_ NP-treated conventional cotton, whose P content was slightly lower relative to its corresponding control group. Fe_2_O_3_ NPs had no impact on B content in roots, regard less of plant types (**Figure 4I**).

### Effects of Fe_2_O_3_ NPs on Hormone Concentration in Cotton Plants

**FIGURE 5:**
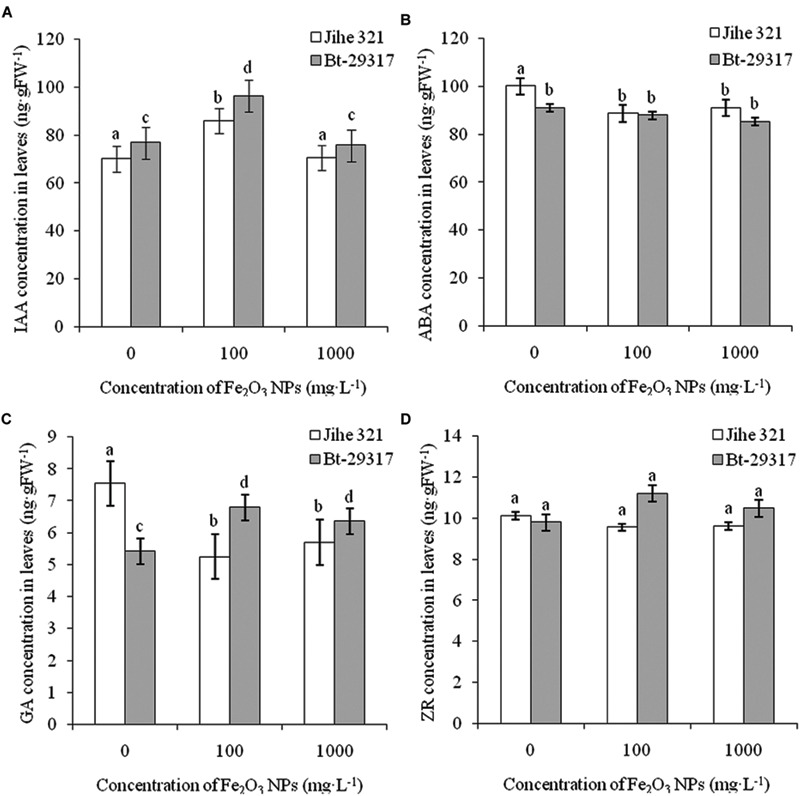
**Effects of Fe_2_O_3_ NPs on hormone concentration in leaves of cotton plants.** The concentrations of IAA, AA, GA, and ZR in Fe_2_O_3_ NPs treated shoots are shown in **A–D**, respectively. The values are presented as means ± SD. Different small letters for the same cultivar indicate a significant difference at *p* < 0.05 level between control and Fe_2_O_3_ NP exposures, and different small letters for the same Fe_2_O_3_ NP concentration indicate a significant difference at *p* < 0.05 level between Bt-transgenic and non-transgenic cotton.

**FIGURE 6:**
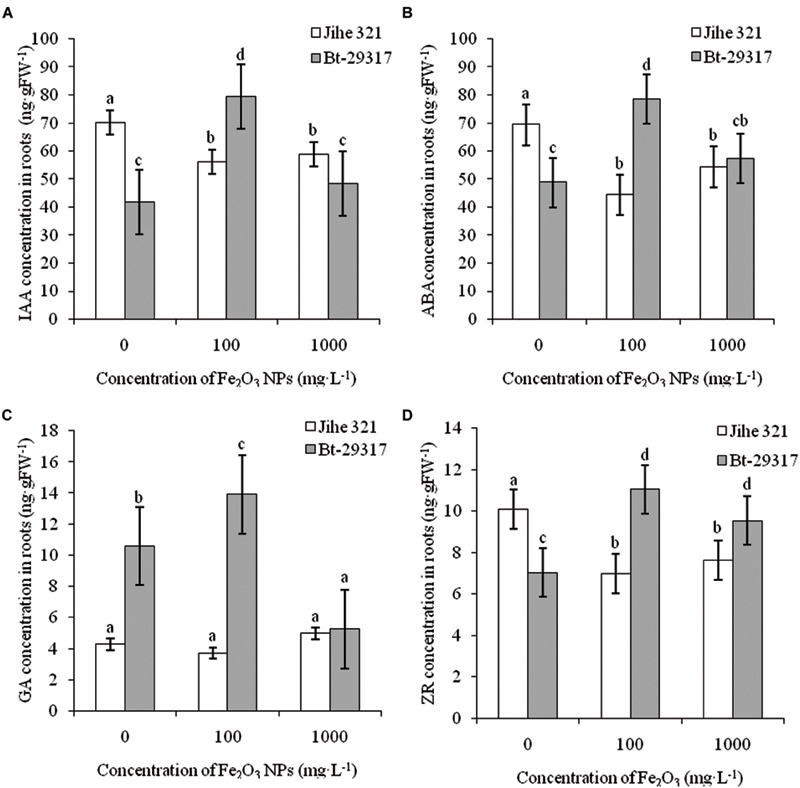
**Effects of Fe_2_O_3_ NPs on hormone concentration in roots of cotton plants.** The concentrations of IAA, AA, GA, and ZR in Fe_2_O_3_ NPs treated roots are shown in **A–D**, respectively. The values are presented as means ± SD. Different small letters for the same cultivar indicate a significant difference at *p* < 0.05 level between control and Fe_2_O_3_ NP exposures, and different small letters for the same Fe_2_O_3_ NP concentration indicate a significant difference at *p* < 0.05 level between Bt-transgenic and non-transgenic cotton.

The effects of Fe_2_O_3_ NPs on plant hormones in conventional and Bt-transgenic cottons are shown in **Figures 5** and **6**. IAA contents in leaves of both types of cottons significantly increased (*p* < 0.05) at an exposure dose of 100 mg⋅L^-1^ Fe_2_O_3_ NPs, but no change was found at 1000 mg⋅L^-1^Fe_2_O_3_ NP treatment (**Figure 5A**). A decrease in ABA concentration was only found in the Fe_2_O_3_ NP-treated conventional cotton, while Fe_2_O_3_ NPs had no impact on ABA concentrations in the leaves of Bt-transgenic cotton. In addition, no significant difference in ABA concentration was seen between Bt-transgenic and non-transgenic cotton leaves under Fe_2_O_3_ NP exposure (**Figure 5B**). Similar results were found for ZR concentration in the leaves of both cotton plants in **Figure 5D**. The GA content in the leaves of conventional cotton declined for both Fe_2_O_3_ NPs treatments, whereas it significantly increased in the leaves of Bt-transgenic cotton (**Figure 5C**).

Fe_2_O_3_ NPs enhanced the concentrations of all four plant hormones (including IAA, ABA, GA, and ZR) in the roots of Bt-transgenic cotton, especially at 100 mg⋅L^-1^ Fe_2_O_3_ NP exposure. However, IAA, ABA, and ZR concentrations in the roots of Fe_2_O_3_ NP-treated conventional cotton significantly decreased. In addition, Fe_2_O_3_ NPs had no impact on GA concentration in the roots of conventional cotton (**Figure 6**). This suggests that the root hormone concentrations in conventional and Bt-transgenic cotton had different responses to Fe_2_O_3_ NP exposure. Le et al. (2014, 2015) reported that IAA and ABA concentrations in the roots of conventional and Bt-transgenic cottons were altered by SiO_2_ and CeO_2_ NPs. Gui et al. (2015) reported that phytohormones such as IAA and ABA intransgenic and non-transgenic rice (*O. sativa*) were affected by 2, 20, and 200 mg⋅L^-1^Fe_2_O_3_ NP exposures. IAA stimulates growth processes such as cell elongation and division, whereas ABA controls plant senescence and responses to stress (Davies, 1995; Mok and Mok, 2001). Thus, our results further demonstrate that NPs could have negative effects on the plant growth process, development, and senescence by manipulating the phytohormone concentration in plants.

### Fe contents in the Shoots and Roots of Cotton Plants

**FIGURE 7:**
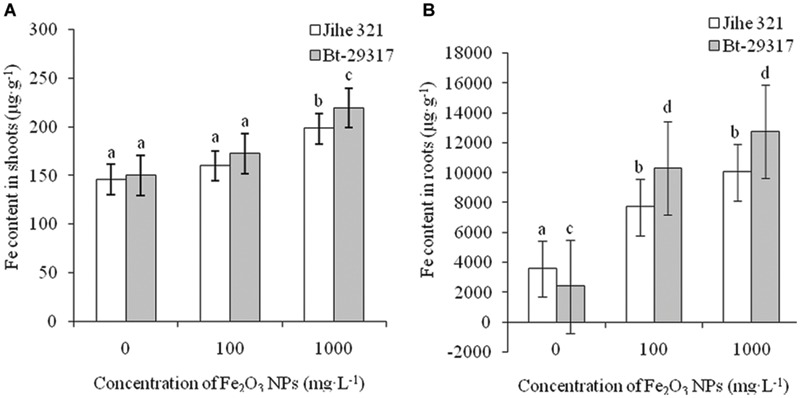
**Fe content in the shoots and roots of cotton plants.** The Fe contents in the shoots and roots of both conventional and Bt-transgenic cottons are shown in **A,B**, respectively. The values are presented as means ± SD. Different small letters for the same cultivar indicate a significant difference at *p* < 0.05 level between control and Fe_2_O_3_ NP exposures, and different small letters for the same Fe_2_O_3_ NP concentration indicate a significant difference at *p* < 0.05 level between Bt-transgenic and non-transgenic cotton.

**Figure 7** shows the total Fe contents in shoots and roots of both conventional and Bt-transgenic cotton treated with two concentrations of Fe_2_O_3_ NPs. No difference was seen in Fe contents in both cotton shoots for treatment with 100 mg⋅L^-1^ Fe_2_O_3_ NPs when compared with the control group. However, as the concentration of Fe_2_O_3_ NPs increased to 1000 mg⋅L^-1^, Fe contents in both cotton varieties were significantly higher than the control groups, and Fe content in the Bt-transgenic cotton was significantly higher than that in conventional cotton (**Figure 7A**). Fe contents in roots of both Fe_2_O_3_ NP-treated cottons are shown in **Figure 7B**. Fe content in the roots of Bt-transgenic cotton treated with 1000 mg⋅L^-1^ Fe_2_O_3_ NPs was 5.30 times higher than in its corresponding control, while this value was 2.8 times for 1000 mg⋅L^-1^ Fe_2_O_3_ NP-treated conventional cotton as compared with its corresponding control. In addition, Fe content in Bt-transgenic cotton roots was 1.27 times higher than that of conventional cotton at 1000 mg⋅L^-1^ Fe_2_O_3_ NP treatment. This indicates that the Bt-transgenic cotton was capable of taking up more Fe relative to the conventional cotton, which agrees with a previous study (Li et al., 2014) in which CeO_2_ NPs aggregates more easily penetrated into the roots of Bt-transgenic cotton than conventional cotton. A small amount of Fe_2_O_3_ NPs was transported to the shoots. Our results are in agreement with previous studies that showed that CuO NPs were taken up by maize through the root system (Wang et al., 2012) and CeO_2_ and SiO_2_ NPs were transported to the shoots from the cotton root system (Le et al., 2014; Li et al., 2014).

**FIGURE 8:**
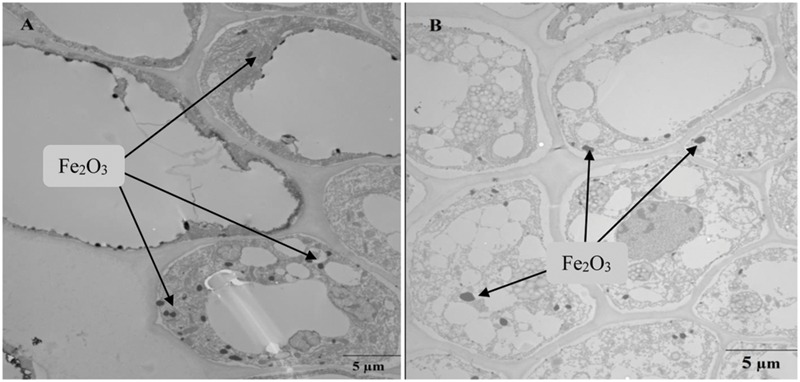
**Transmission electron microscopy images of root sections of non-transgenic cotton **(A)** and Bt-transgenic cotton **(B)** plants after 10 days of treatment with Fe_2_O_3_ NPs**.

Transmission electron microscopy images of the root sections of Bt-transgenic and non-transgenic cotton show the presence of dark dots (particles) primarily localized in the endodermis and vascular cylinder with 1000 mg⋅L^-1^ Fe_2_O_3_ NP exposure (**Figure 8**). The absorption of Fe_2_O_3_ NPs and their aggregation in the roots of both conventional and Bt-transgenic cottons are evident. Most Fe_2_O_3_ NPs were found in the root outer epidermis, and only a few NPs were localized in the intercellular spaces. Thus, it can be concluded that the Fe_2_O_3_ NPs could enter the endodermis and vascular cylinder of both Bt-transgenic and non-transgenic cotton. Previous studies also demonstrated that SiO_2_ and CeO_2_ NPs were localized in the intercellular spaces of both types of cotton (Le et al., 2014; Li et al., 2014). ZnO NPs were also observed in the endodermis and vascular cylinder of ryegrass roots (Lin and Xing, 2008).

### Effects of Fe_2_O_3_ NPs on Bt Toxin in Bt-Transgenic Cotton

**FIGURE 9:**
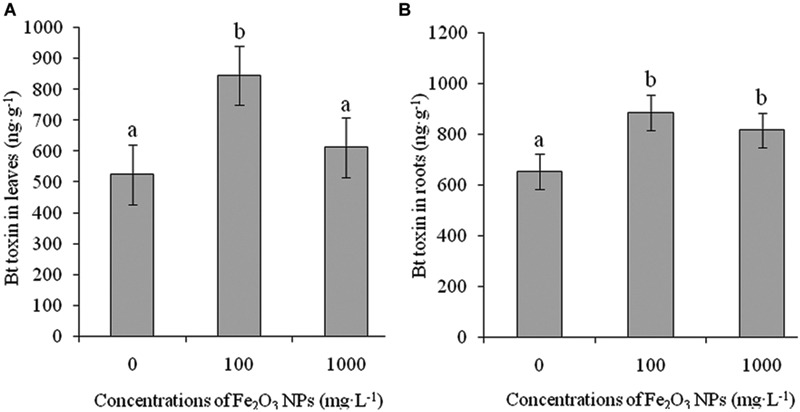
**Effects of Fe_2_O_3_ NPs on Bt toxin in leaves and roots of Bt-transgenic cotton. (A,B)** Represents the levels of Bt toxin in the shoots and roots of transgenic cotton. The values are presented as means ± SD. Different small letters for the same cultivar indicate a significant difference at *p* < 0.05 level between control and Fe_2_O_3_ NP exposure.

Adamczyk and Sumerford (2001) reported that the performance of *Bt* genes for controlling target insect pests varies according to cotton variety, age of plant (Wan et al., 2005), part of plant (Abel and Adamczyk, 2004), type of gene, and insertion site of the gene into the DNA of target plants (Gore et al., 2001; Gore and Adamczyk, 2004; Jackson et al., 2004). Bt toxin is the product of an exogenous Bt gene, whose concentration in shoots is the most important index for evaluating the insect resistant ability of Bt cotton; however, if the root expresses more Bt toxin, it can negatively affect the soil ecological system (Saxena et al., 1999; Obrycki et al., 2001; Fu et al., 2012). **Figure 9** shows the expression of Bt toxin in leaves and roots of Bt-transgenic cotton in the presence of Fe_2_O_3_ NPs. A significant difference in the contents of Bt toxin was seen in both leaves and roots of Bt-transgenic cotton treated with various concentrations of Fe_2_O_3_ NPs as compared with the control group. Bt toxin concentrations in leaves and roots were 845.89 and 886.94 ng⋅g^-1^, respectively, at the exposure dose of 100 mg⋅L^-1^ Fe_2_O_3_ NPs, which were 1.61 and 1.36 times higher than their respective control group. Upon exposure to 1000 mg⋅L^-1^ Fe_2_O_3_ NPs, the Bt toxin in Bt-transgenic cotton leaves and roots were still greater (*p* < 0.05) than that of the control group, although they were lower than in the lower exposure dose of Fe_2_O_3_ NP-treated Bt-transgenic cotton. The expression of Bt toxin in Bt-transgenic cotton leaves increased, especially at 100 mg⋅L^-1^ Fe_2_O_3_ NPs, which could play an important role in resisting insect damage. To reduce the risk of resistance development in target insect pests against Bt cotton and agricultural crops, there is a need to understand variations in the efficiency of Bt genes and the application of NPs in agriculture.

## Conclusion

The present study demonstrated that Fe_2_O_3_ NPs could inhibit the plant height and root length of Bt-transgenic cotton, as well as promote the root hairs and biomass of conventional cotton. The effects of Fe_2_O_3_ NPs on nutrient contents in the shoots and roots of both types of cotton were investigated. Fe_2_O_3_ NPs enhanced Na content in the roots of Bt-transgenic plants, and similar results were observed for K contents in the roots of both Bt-transgenic and non-transgenic cotton. Zn contents in the roots of Bt-transgenic cotton decreased upon Fe_2_O_3_ NP exposure, while Cu contents in the roots of both types of cotton increased upon exposure to 100 mg⋅L^-1^Fe_2_O_3_ NPs and decreased at 1000 mg⋅L^-1^ Fe_2_O_3_ NPs. Responses of hormone concentrations in the presence of Fe_2_O_3_ NPs differed between the leaves and roots of both types of cottons. Most hormones in the roots of Bt-transgenic cotton increased at low Fe_2_O_3_ NP exposure (100 mg⋅L^-1^), but decreased at a high concentration of Fe_2_O_3_ NPs (1000 mg⋅L^-1^). In addition, Bt-toxin in the leaves and roots of both Bt-transgenic and non-transgenic cotton increased upon NP exposure. TEM images shows that Fe_2_O_3_ NPs were evident in the root sections of both Bt-transgenic and non-transgenic cotton, and Fe contents in the shoots and roots increased with increasing exposure doses of Fe_2_O_3_ NPs. The present study illustrates the bioaccumulation of Fe_2_O_3_ NPs in plants, which might have potential risks for agricultural crops and affect the environment and human health.

## Author Contributions

LN and YR conceived and designed the experiment. CM provided scientific expertise. LN, CM, YR, WC, YD, LL, and BX performed the experiments and analyzed the data. LN, CM, and YR wrote and revised the paper. All authors have read and approved the final manuscript.

## Conflict of Interest Statement

The authors declare that the research was conducted in the absence of any commercial or financial relationships that could be construed as a potential conflict of interest.

## Abbreviations

ABA: abscisic acid
DI H_2_O: deionized water
GA: gibberellic acid
h: hours
IAA: indole-3-acetic acid
NPs: Nanoparticles
SD: standard deviation
TEM: transmission electron microscopy
ZR: trans-zeatin riboside.
